# In vitro induction of tetraploidy and its effects on phenotypic variations in *Populus hopeiensis*

**DOI:** 10.1186/s12870-023-04578-0

**Published:** 2023-11-13

**Authors:** Jian Wu, Qing Zhou, Yaru Sang, Yifan Zhao, Bo Kong, Liang Li, Jiahua Du, Lexun Ma, Min Lu, Pingdong Zhang

**Affiliations:** 1 Key Laboratory for Forest Resources Conservation and Utilization in the Southwest Mountains of China, Ministry of Education, Southwest Forestry University, Kunming, 650224 China; 2 State Key Laboratory of Efficient Production of Forest Resource, Beijing Forestry University, Beijing, 100083 China; 3 Key Laboratory of Genetics and Breeding in Forest Trees and Ornamental Plants, Ministry of Education, Beijing Forestry University, Beijing, 100083 China; 4 College of Biological Sciences and Technology, Beijing Forestry University, Beijing, 100083 China; 5 Institute of Genetics and Breeding, Inner Mongolia Academy of Forestry, Hohhot, 010010 China

**Keywords:** Tetraploid, Colchicine, Stomata, Phenotypic variation, *Populus hopeiensis*

## Abstract

**Background:**

Artificial induction of polyploidy is the most common and effective way to improve the biological properties of *Populus* and develop new varieties of this tree*.* In this study, in order to confirm and expand earlier findings, we established a protocol using colchicine and based on an efficient shoot regeneration system of leaf blades to induce tetraploidy in vitro in three genotypes from diploid *Populus hopeiensis*. The stomatal characteristics, leaf blade size, and growth were evaluated for diploids and tetraploids of three genotypes.

**Results:**

We found that genotype, preculture duration, colchicine concentration, and colchicine exposure time had highly significant effects on the tetraploid induction rate. The optimal protocol for inducing tetraploidy in *P. hopeiensis* was to preculture leaf blades for 7 days and then treat them for 4 days with 40 mg/L colchicine. The tetraploid induction rates of genotypes BT1, BT3, and BT8 were 21.2, 11.4 and 16.7%, respectively. A total of 136 tetraploids were identified by flow cytometry analysis and somatic chromosome counting. The stomatal length, width, and density of leaf blades significantly differed between diploid and tetraploid plants. Compared with their diploid counterparts, the tetraploids produced larger leaf blades and had a slower growth rate. Our findings further document the modified morphological characteristics of *P. hopeiensis* following whole-genome duplication (e.g., induced tetraploidy).

**Conclusions:**

We established a protocol for in vitro induction of tetraploidy from diploid leaf blades treated with colchicine, which can be applied to different genotypes of *P. hopeiensis*.

## Background

Triploid breeding is one of the most effective approaches for *Populus* genetic improvement. For example, growth rates [1, 2], fiber properties [3, 4], timber quality [5, 6], and biomass production [7–9] have been enhanced using a triploid breeding program of *Populus*. The improved performance of triploids is due to the combined effect of higher ploidy levels and heterosis [10]. The most direct method to achieve new triploid germplasms is to cross diploids with tetraploids [11–13]. However, the application of this method has been limited due to the lack of natural tetraploids. Therefore, artificial tetraploids induced by colchicine are useful as bridge parents to produce the triploids in *Populus.*

In vascular plants, somatic chromosome doubling has already been successfully implemented to generate new germplasms with higher ploidy levels. Woody perennials such as octaploid *Ziziphus jujuba* [14], *Neolamarckia cadamba* [15], *Jatropha curcas* [16], and *Panicum virgatum* L. [17, 18]; and hexaploid *Populus* [19, 20], *Pennisetum* [21, 22], and *Prunus pseudocerasus* [23], as well as tetraploid *Populus* [24–26], *Ziziphus jujuba* [27, 28], *Robinia pseudoacacia* [29, 30], *Platanus acerifolia* [31], *Pogostemon cablin* [32], *Liquidambar styraciflua* [33], and *Lagerstroemia indica* [34] are examples of successful colchicine induction to achieve somatic chromosome doubling. Different explant types in vascular plants have been induced to obtain somatic chromosome doubling: the explant types include seeds [35], zygotes [36–38], shoot tips [16, 30, 39], nodal segments [15, 20], leaf petioles [33, 40], and leaf blades [24, 28]. Hence, somatic chromosome doubling is a promising method to produce new polyploids.

Increase in the chromosome number of tetraploid plants is actually the result of whole-genome duplication events. It is worth mentioning that Populous in general (while currently diploid) has evidence of a whole genome duplication event [41, 42]. One of the most interesting features of whole-genome duplication is the increased dosage of all genes, which alters gene expression resulting in increased cell and organelle sizes, and ultimately, variation in the traits of tetraploid plants [43, 44]. Furthermore, whole-genome duplication events often confer tetraploid plants with superior traits compared to their isogenic diploid progenitor. For example, faster growth [28], larger leaf blades [24, 26, 32], thicker leaf blades [29, 31, 45], thicker stems [46, 47], larger flowers [27, 48, 49], and greater numbers of seeds [50] are often found in synthetic tetraploid plants. Additionally, several studies have shown that tetraploidization improves physiological tolerance to stress in several plant species, such as *Ziziphus jujuba* [51], *Escallonia* [49], *Stevia rebaudiana* [52], *Dioscorea zingiberensis* [53], *Dendranthema nankingense* [54]. Thus, tetraploidy is thought to play an important role in plant diversification and evolution.

Here, we focused on the model woody plant *Populus* (*Populus hopeiensis*) to investigate the phenotypic consequences of gene dosage effects. For this, a protocol for in vitro tetraploid induction of *P. hopeiensis* by colchicine treatment was established based on an efficient shoot regeneration system using leaf blades. To compare the different ploidy levels of the three genotypes, the responses of stomatal characteristics, leaf blade size, and growth traits were evaluated. Our data showed that tetraploids of *P. hopeiensis* were distinguished from their isogenic diploid progenitor by having larger stomata, larger leaf blades, and a slower growth rate.

## Results

### Shoot regeneration of leaf blades

**Fig. 1:**
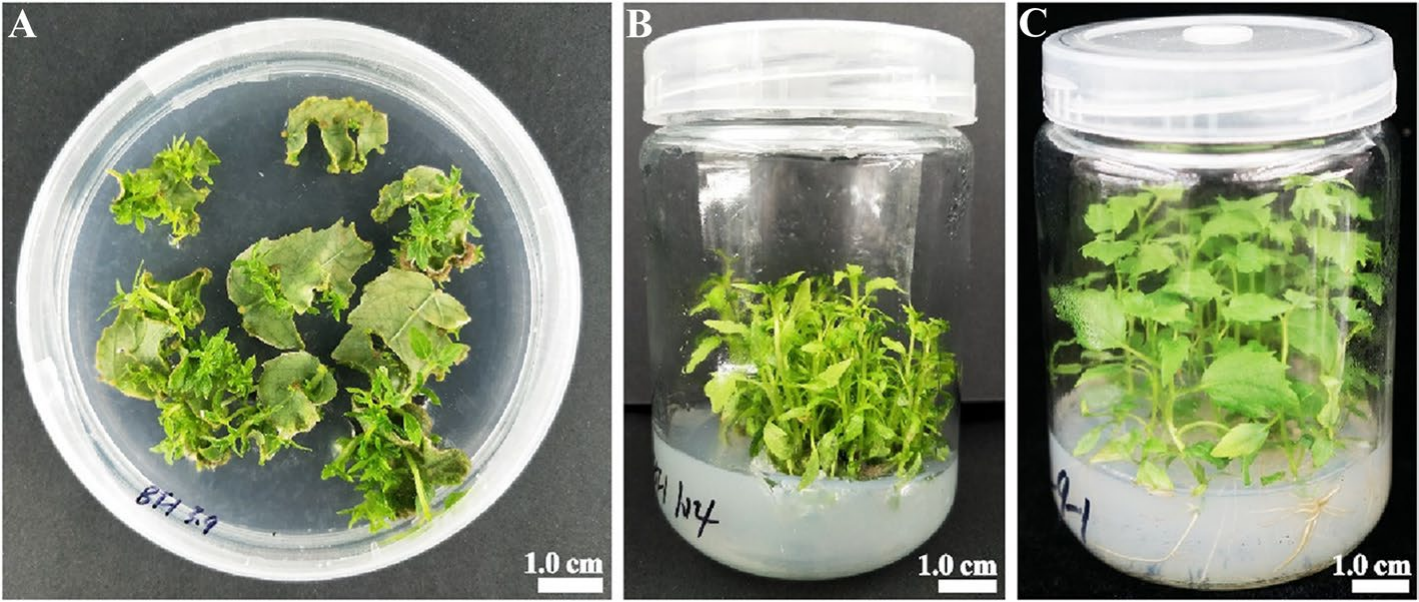
The regeneration of plants from leaf blades of *P. hopeiensis*. **A** Shoots formed from leaf blades after culture for 25 days. **B** Adventitious shoots regenerated from leaf blades after culture for 45 days. **C** Successfully rooted healthy regenerated plants

Nine treatments were used to evaluate the effects of 6-BA, TDZ, and IAA on shoot regeneration from *P. hopeiensis* leaf blades. Adventitious shoots formed from leaf blades after 25 days of culture on solid shoot regeneration medium (Fig. 1A), and healthy adventitious shoots successfully regenerated from leaf blades after 45 days (Fig. 1B). The roots of regenerated plants were formed after 30 days of culture on rooting medium (Fig. 1C). The shoot regeneration rates from nine treatments are shown in Table 1. The shoot regeneration rates ranged from 16.7 to 96.7%. The univariate generalized linear model (GLM) analysis showed that genotype (F = 7.190, *P* = 0.001), 6-BA (F = 84.451, *P* = 0.000), TDZ (F = 21.486, *P* = 0.000), and IAA (F = 25.062, *P* = 0.000) had highly significant effects on the shoot regeneration rates. Least significant difference (LSD) multiple comparison tests revealed that the shoot regeneration rate of genotype BT1 was significantly higher than that of genotypes BT3 or BT8 (*P* < 0.05) (Table 2). The shoot regeneration rate was significantly higher on the medium containing 0.4 mg/L 6-BA than those on medium containing 0.3 or 0.2 mg/L 6-BA. The shoot regeneration rate was also significantly higher on medium containing 0.015 mg/L TDZ than those on medium containing 0.01 or 0.005 mg/L TDZ. However, the shoot regeneration rate was significantly lower on media containing 0.2 mg/L IAA than those on medium containing 0.1 or 0.05 mg/L IAA.

**Table 1:** Effects of 6-BA, TDZ, and IAA concentrations on shoot regeneration from leaf blades representing three genotypes of *P. hopeiensis*

Treatment	Hormone concentrations (mg/L)			Shoot regeneration rate (%)			Mean	No. of shoots per explant			Mean
	6-BA	TDZ	IAA	BT1	BT3	BT8		BT1	BT3	BT8	
1	0.2	0.005	0.05	16.7 ± 5.8	20.0 ± 10.0	33.3 ± 5.8	23.3 ± 10.0	2.0 ± 0.5	3.1 ± 0.6	3.3 ± 0.3	2.8 ± 0.7
2	0.2	0.010	0.10	30.0 ± 10.0	26.7 ± 5.8	23.3 ± 5.8	26.7 ± 7.1	4.3 ± 0.3	5.1 ± 0.6	4.1 ± 0.6	4.5 ± 0.6
3	0.2	0.015	0.20	23.3 ± 5.8	16.7 ± 5.8	20.0 ± 10.0	20.0 ± 7.1	3.6 ± 0.5	2.2 ± 0.8	1.2 ± 0.4	2.3 ± 1.1
4	0.3	0.005	0.10	53.3 ± 5.8	30.0 ± 10.0	36.7 ± 5.8	40.0 ± 12.2	4.5 ± 0.4	6.2 ± 0.5	4.4 ± 0.3	5.1 ± 0.9
5	0.3	0.010	0.20	40.0 ± 10.0	36.7 ± 5.8	30.0 ± 10.0	35.6 ± 8.8	3.4 ± 0.3	4.5 ± 0.6	3.0 ± 0.5	3.6 ± 0.8
6	0.3	0.015	0.05	70.0 ± 10.0	83.3 ± 5.8	60.0 ± 10.0	71.1 ± 12.7	7.8 ± 0.8	6.7 ± 0.4	5.9 ± 0.4	6.8 ± 1.0
7	0.4	0.005	0.20	50.0 ± 10.0	36.7 ± 5.8	43.3 ± 5.8	43.3 ± 8.7	5.4 ± 0.2	5.2 ± 1.5	3.7 ± 0.7	4.8 ± 1.2
8	0.4	0.010	0.05	86.7 ± 15.3	70.0 ± 10.0	66.7 ± 15.3	74.4 ± 15.1	8.0 ± 0.4	7.7 ± 0.3	7.9 ± 1.3	7.9 ± 0.7
9	0.4	0.015	0.10	96.7 ± 5.8	80.0 ± 10.0	63.3 ± 5.8	80.0 ± 15.8	11.7 ± 1.5	9.3 ± 0.5	8.4 ± 0.2	9.8 ± 1.7

The data represent the mean ± SE of three replicates. Genotype BT1, BT3 and BT8 are cultivars

**Table 2:** Shoot regeneration rate and the number of shoots per explant according to genotype and different hormones at a range of concentrations

Treatment	Levels	Shoot regeneration rate (%)	No. of shoots per explant
Genotype	BT1	51.9 ± 27.9a	5.6 ± 3.0a
	BT3	44.4 ± 25.9b	5.5 ± 2.2a
	BT8	41.9 ± 18.4b	4.7 ± 2.3b
6-BA (mg/L)	0.2	23.3 ± 8.3c	3.2 ± 1.3c
	0.3	48.9 ± 19.5b	5.2 ± 1.6b
	0.4	65.9 ± 21.0a	7.5 ± 2.4a
TDZ (mg/L)	0.005	35.6 ± 13.4c	4.2 ± 1.4c
	0.01	45.6 ± 23.6b	5.3 ± 2.0b
	0.015	57.0 ± 29.5a	6.3 ± 3.4a
IAA (mg/L)	0.05	56.3 ± 26.8a	5.8 ± 2.4b
	0.1	48.9 ± 25.9a	6.4 ± 2.7a
	0.2	33.0 ± 12.7b	3.6 ± 1.4c

The data represent the mean ± SE. The different letters indicate significant differences based on the LSD at the 0.05 probability level

The number of shoots per explant from nine treatments are presented in Table 1. The number of shoots per explant varied from 1.2 to 11.7. The number of shoots per explant significantly varied among different genotypes (F = 9.268, *P* = 0.000) and among the hormone treatments—6-BA (F = 148.470, *P* = 0.000), TDZ (F = 34.747, *P* = 0.000), and IAA (F = 72.259, *P* = 0.000)—according to univariate GLM analysis. The LSD multiple comparison tests showed that the number of shoots per explant of genotypes BT1 or BT3 was significantly higher than that of genotype BT8 (*P* < 0.05) (Table 2). The number of shoots per explant was significantly higher on the medium containing 0.4 mg/L 6-BA than those on medium containing 0.3 or 0.2 mg/L 6-BA. The number of shoots per explant was also significantly higher on medium containing 0.015 mg/L TDZ than those on medium containing 0.01 or 0.005 mg/L TDZ. However, the number of shoots per explant was significantly lower on medium containing 0.2 or 0.05 mg/L IAA than those on medium containing 0.1 mg/L IAA. Based on these data, Murashige and Skoog (MS) medium containing 0.4 mg/L 6-BA, 0.015 mg/L TDZ, and 0.01 mg/L IAA was determined to be suitable for shoot regeneration from leaf blades of *P. hopeiensis*.

### Tetraploid induction of leaf blades treated with colchicine

**Fig. 2:**
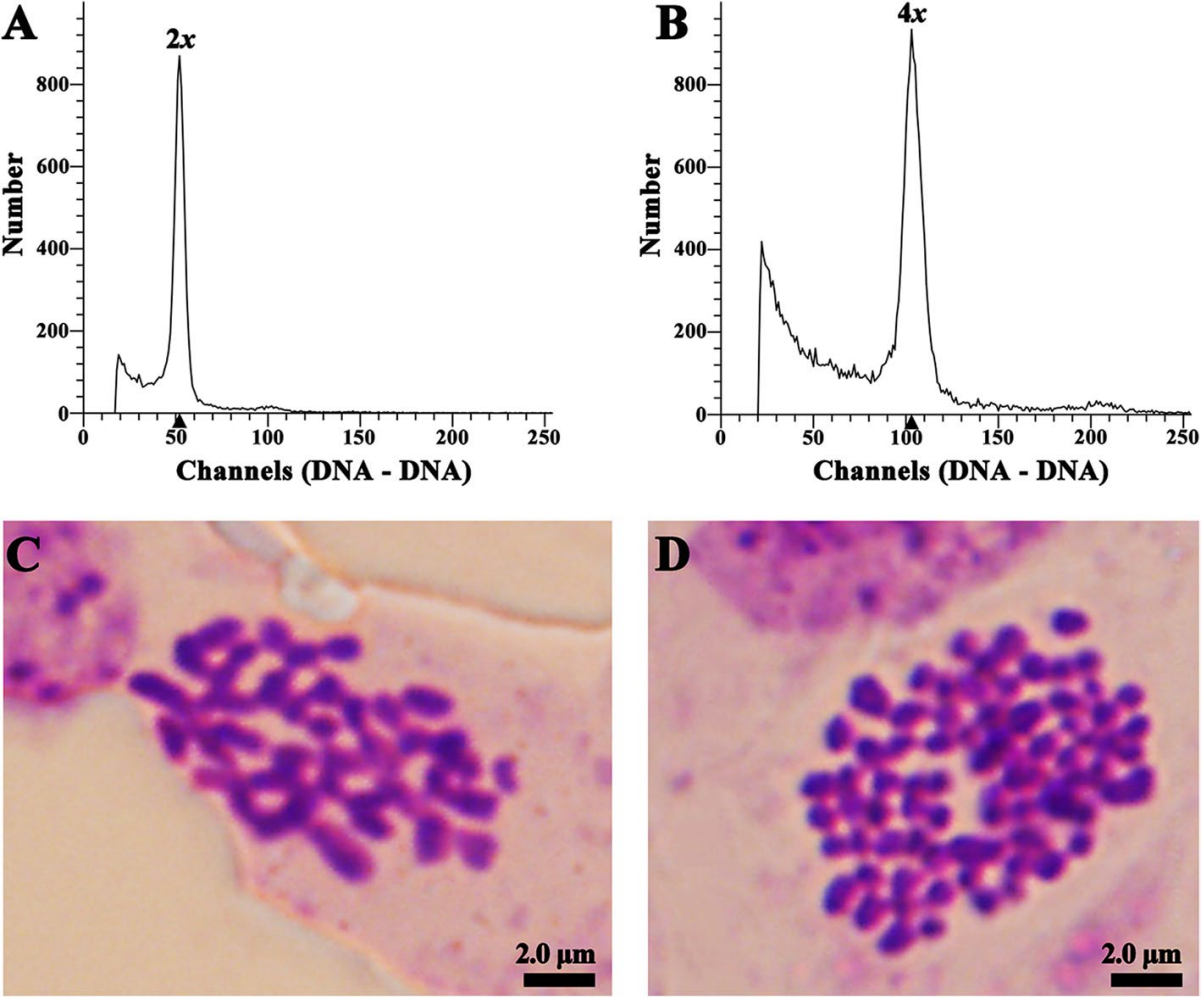
Determination of the ploidy levels of regenerated *P. hopeiensis* plants. **A** Flow cytometry histogram of the diploids. **B** Flow cytometry histogram of the tetraploids. **C** Somatic chromosome number of the diploids (2n = 2*x* = 38). **D** Somatic chromosome number of the tetraploids (2n = 4*x* = 76)

A total of 4,736 regenerated plants of the three genotypes were obtained from 27 treatments after leaf blades were treated with colchicine (Table 3). Individual regenerated plants were classified as diploid (Fig. 2A) or tetraploid (Fig. 2B) based on the peaks obtained by flow cytometry analysis. A total of 136 putative tetraploids were preliminarily determined. Somatic chromosome counting showed that the chromosome number of diploids was 2n = 2*x* = 38 (Fig. 2C), and all the putative tetraploids were ultimately confirmed to be real tetraploids (2n = 4*x* = 76, Fig. 2D).

**Table 3:** Effect of preculture duration and colchicine concentration and exposure time on somatic cell chromosome doubling of *P. hopeiensis*

Treatment	Preculture duration (d)	Colchicine concentration (mg/L)	Exposure time (d)	No. of shoots regenerated^a^			No. of tetraploid^b^			Tetraploid induction rates (%)^c^		
				BT1	BT3	BT8	BT1	BT3	BT8	BT1	BT3	BT8
1	5	20	2	102	96	91	1	0	0	0.9 ± 1.6	0.0 ± 0.0	0.0 ± 0.0
2	5	20	3	80	68	67	2	0	5	3.0 ± 2.6	0.0 ± 0.0	7.9 ± 3.7
3	5	20	4	40	53	56	0	0	6	0.0 ± 0.0	0.0 ± 0.0	10.4 ± 1.9
4	5	30	2	75	85	68	0	0	4	0.0 ± 0.0	0.0 ± 0.0	5.7 ± 1.5
5	5	30	3	55	74	49	0	1	0	0.0 ± 0.0	1.0 ± 1.7	0.0 ± 0.0
6	5	30	4	25	47	27	1	0	0	2.8 ± 4.8	0.0 ± 0.0	0.0 ± 0.0
7	5	40	2	51	59	35	4	0	0	7.6 ± 1.7	0.0 ± 0.0	0.0 ± 0.0
8	5	40	3	32	40	15	0	0	0	0.0 ± 0.0	0.0 ± 0.0	0.0 ± 0.0
9	5	40	4	16	22	9	1	0	0	4.2 ± 7.2	0.0 ± 0.0	0.0 ± 0.0
10	6	20	2	106	110	119	2	0	4	1.9 ± 1.7	0.0 ± 0.0	3.3 ± 1.1
11	6	20	3	79	87	86	2	0	6	2.8 ± 2.8	0.0 ± 0.0	6.8 ± 1.7
12	6	20	4	57	80	69	0	0	12	0.0 ± 0.0	0.0 ± 0.0	17.8 ± 2.0
13	6	30	2	92	78	69	1	0	0	1.0 ± 1.7	0.0 ± 0.0	0.0 ± 0.0
14	6	30	3	63	68	53	1	0	2	1.7 ± 2.9	0.0 ± 0.0	3.3 ± 3.0
15	6	30	4	49	57	50	7	2	4	14.4 ± 0.9	3.0 ± 2.7	8.5 ± 3.0
16	6	40	2	77	66	51	2	1	0	2.7 ± 2.4	1.7 ± 2.9	0.0 ± 0.0
17	6	40	3	51	56	35	0	1	1	0.0 ± 0.0	1.5 ± 2.5	1.9 ± 3.2
18	6	40	4	28	38	31	0	5	2	0.0 ± 0.0	13.0 ± 2.2	5.6 ± 4.9
19	7	20	2	88	69	86	0	0	1	0.0 ± 0.0	0.0 ± 0.0	0.8 ± 1.4
20	7	20	3	71	60	63	1	0	2	1.2 ± 2.1	0.0 ± 0.0	2.6 ± 2.2
21	7	20	4	65	44	52	0	0	2	0.0 ± 0.0	0.0 ± 0.0	3.4 ± 3.0
22	7	30	2	78	49	65	6	0	0	7.5 ± 3.4	0.0 ± 0.0	0.0 ± 0.0
23	7	30	3	64	39	53	1	1	1	1.4 ± 2.4	2.0 ± 3.4	1.6 ± 2.7
24	7	30	4	44	32	44	2	1	2	3.6 ± 3.2	3.0 ± 5.2	4.3 ± 3.8
25	7	40	2	71	38	58	4	0	3	4.8 ± 4.2	0.0 ± 0.0	5.2 ± 0.4
26	7	40	3	66	30	39	3	1	3	3.9 ± 3.5	3.0 ± 5.2	8.3 ± 2.8
27	7	40	4	62	27	37	13	3	6	21.2 ± 2.0	11.4 ± 2.5	16.7 ± 3.3
Total	-	-	-	1687	1572	1477	54	16	66	-	-	-

^ab^Data represent the sum of three replicates

^c^Data represent the mean ± SE of three replicates

The effects of genotype, preculture duration, colchicine concentration, and colchicine exposure times on the tetraploid induction rates of *P. hopeiensis* leaf blades were investigated (Table 3). The tetraploid induction rates ranged from 0 to 21.2%. The univariate GLM analysis revealed that genotype (F = 30.379, *P* = 0.000), preculture duration (F = 22.869, *P* = 0.000), colchicine concentration (F = 17.068, *P* = 0.000), and colchicine exposure time (F = 65.220, *P* = 0.000) had highly significant effects on the tetraploid induction rate. LSD multiple comparison tests showed that the tetraploid induction rate of genotype BT1 or BT3 was significantly lower than that of genotype BT8 (*P* < 0.05) (Table 4). The tetraploid induction rate following a preculture of 7 or 6 days was significantly higher than that following a preculture of 5 days. The tetraploid induction rate following treatment with 40 mg/L colchicine was significantly higher than that following treatment with 20 or 30 mg/L colchicine. The tetraploid induction rate following treatment with colchicine for 4 days was significantly higher than that following treatment for 2 or 3 days. Hence, the optimal protocol for inducing tetraploid *P. hopeiensis* was preculture of the leaf blades for 7 days, followed by treatment with 40 mg/L colchicine for 4 days.

**Table 4:** Multiple comparisons of tetraploid induction rates derived from different treatments

Treatment	Levels	Tetraploid induction rates (%)
Genotype	BT1	3.2 ± 5.2b
	BT3	1.5 ± 3.6c
	BT8	4.2 ± 5.2a
Preculture duration (d)	5	1.6 ± 3.4b
	6	3.4 ± 5.0a
	7	3.9 ± 5.6a
Colchicine concentration (mg/L)	20	2.3 ± 4.3b
	30	2.4 ± 3.9b
	40	4.2 ± 6.0a
Colchicine exposure time (d)	2	1.6 ± 2.7b
	3	2.0 ± 3.1b
	4	5.3 ± 6.8a

The data represent the mean ± SE. The different letters indicate significant differences based on the LSD at the 0.05 probability level

### Variations in stomata and morphological features in diploid and tetraploid plants

**Fig. 3:**
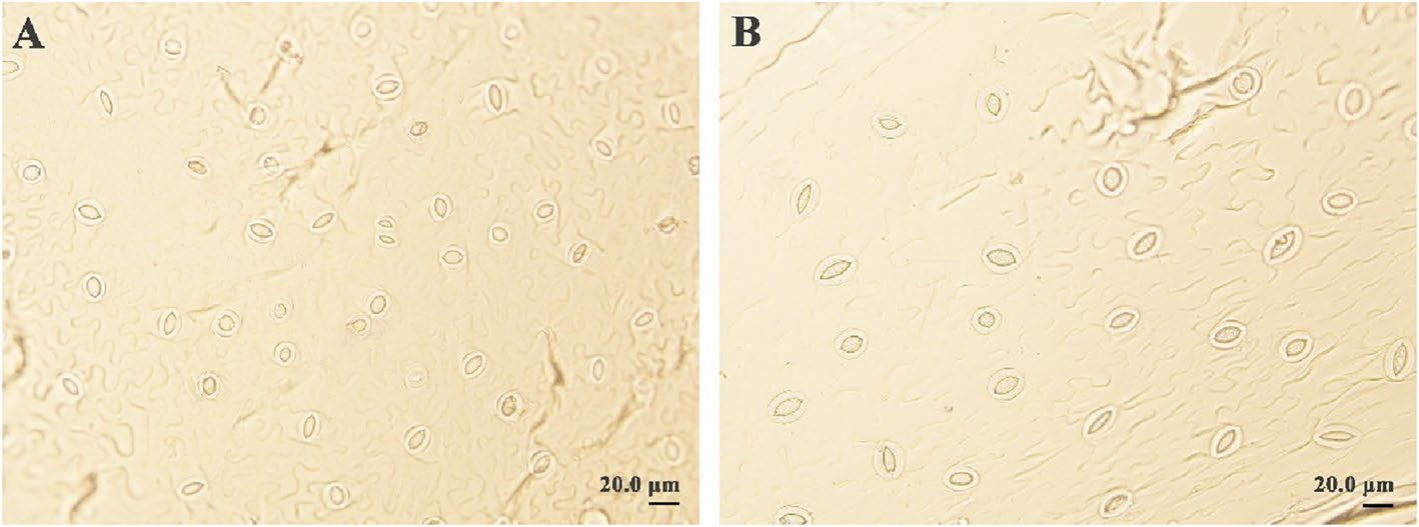
Stomata of the leaf blade in diploid (**A**) and tetraploid (**B**) plants of *P. hopeiensis*

**Fig. 4:**
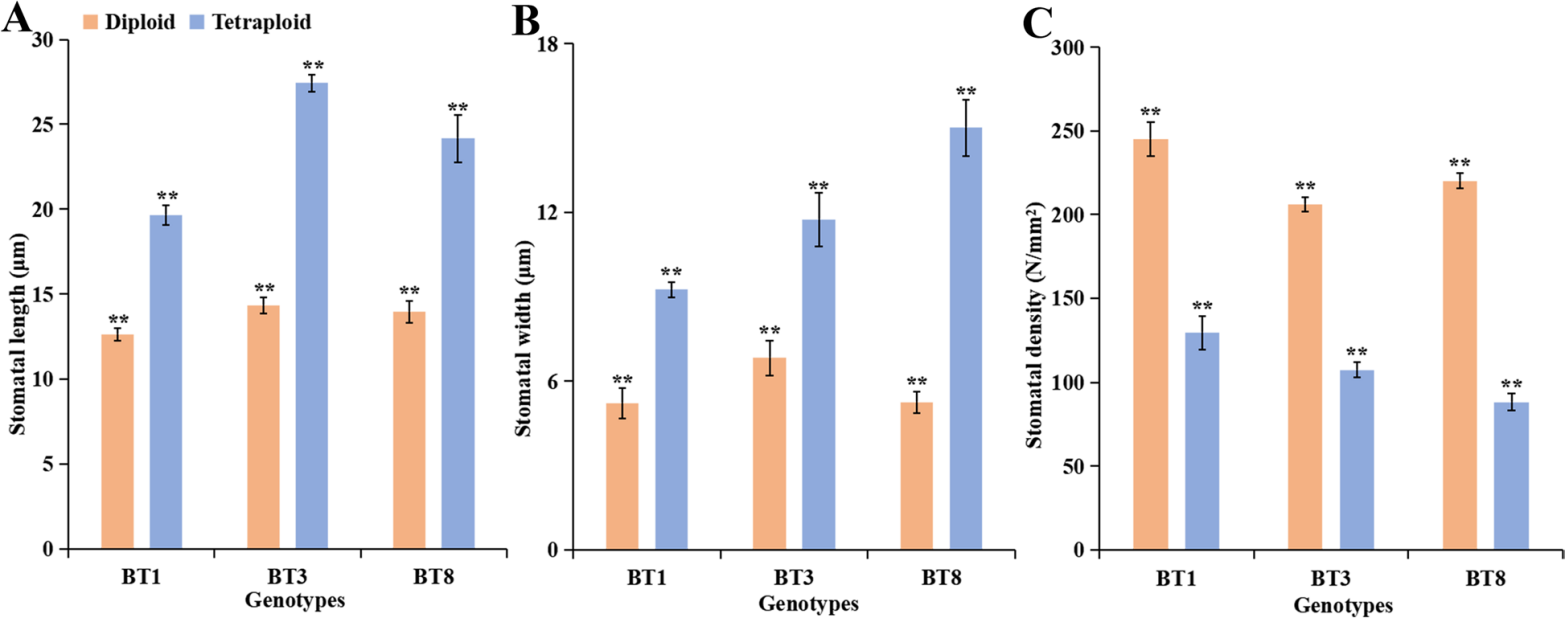
The stomatal length (**A**), stomatal width (**B**), and stomatal density (**C**) in diploid and tetraploid plants of *P. hopeiensis*. The vertical bars show the standard error; the asterisk indicates significant differences between diploid and tetraploid plants (** *p* < 0.01)

To assess the effect of ploidy level on leaf stomata, the lower epidermis of the leaf blades from 30-day-old sterile-rooted plantlets were used for stomata characteristic analysis. As shown in Fig. 3, the leaf stomata size was larger in tetraploids than in their diploid counterparts. The stomatal length, width, and density of diploid and tetraploid plants were measured (Fig. 4). The mean stomatal length and width in tetraploid plants of the three genotypes were significantly higher than that of diploids (Fig. 4A, B) (*t-*test, *P* < 0.01). The stomatal length and width of tetraploid plants were approximately twice that of diploid plants. However, the stomatal density in tetraploids of the three genotypes was significantly lower than that of diploids (Fig. 4C) (*t-*test, *P* < 0.01). The stomatal density of tetraploid plants was about half that of diploid plants. The results imply that stomatal characteristics are useful for distinguishing plant ploidy levels.

**Fig. 5:**
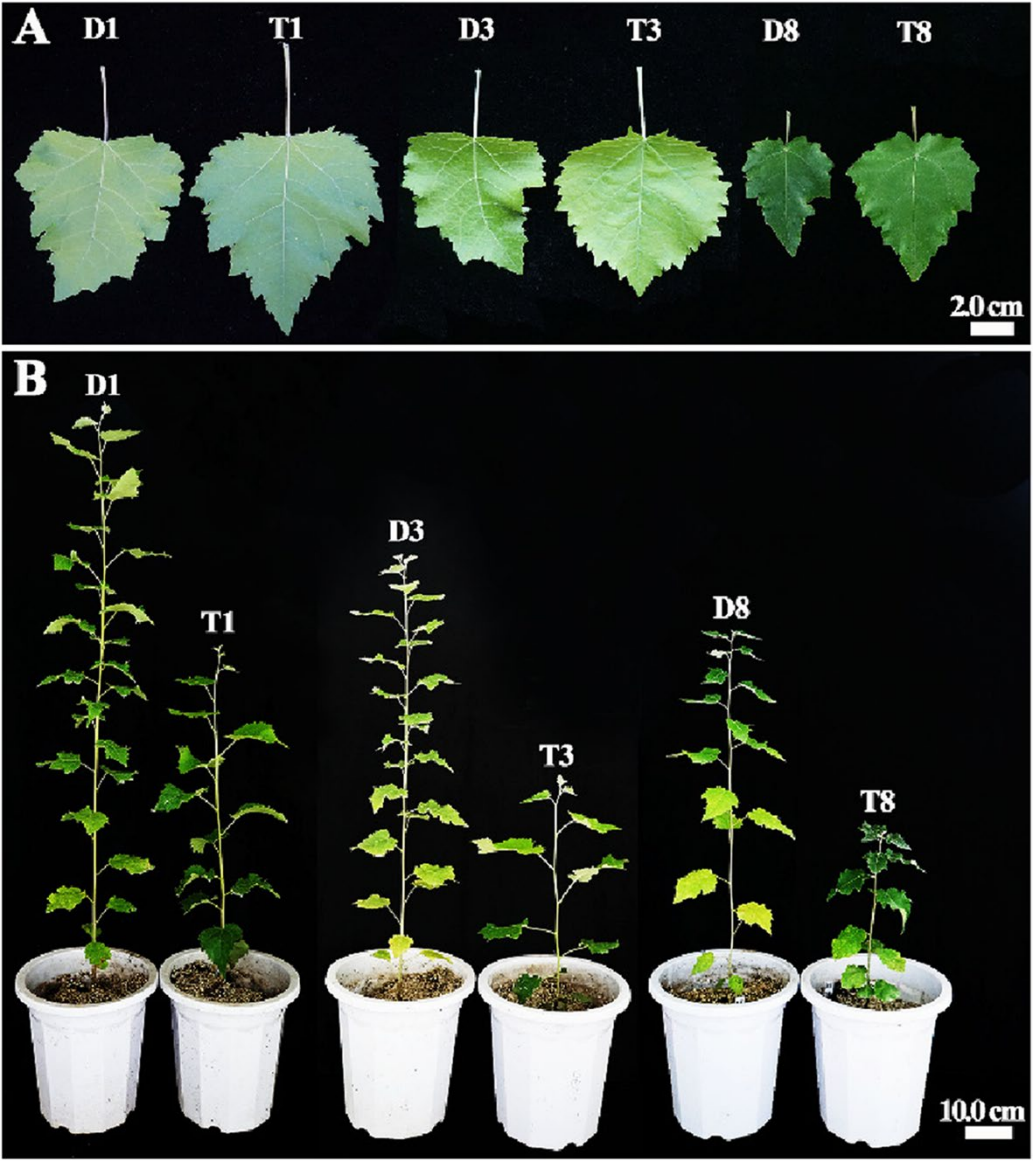
Morphological features in diploid and tetraploid plants of *P. hopeiensis*. **A** Fully expanded leaf blades of diploids and tetraploids. **B** Transplants of regenerated diploids and tetraploids. D1, D3, and D8 represent the diploids of genotypes BT1, BT3, and BT8, respectively. T1, T3, and T8 represent the tetraploids of genotypes BT1, BT3, and BT8, respectively

To analyze the effect of ploidy level on phenotypic variation, 45 diploids and 45 tetraploids for each genotype were transplanted into plastic pots and transferred to greenhouse cultures for 6 months. Changes in the phenotypic traits of diploids and tetraploids of the three genotypes were characterized. The size of the leaf blades was much larger in tetraploid plants than in diploid plants (Fig. 5A). However, due to their slower growth rate, average plant height was lower in the six-month-old tetraploids than in the diploids (Fig. 5B). Pronounced differences in growth were observed among the three genotypes; the growth of the ramets of genotype BT1 was the fastest.

**Fig. 6:**
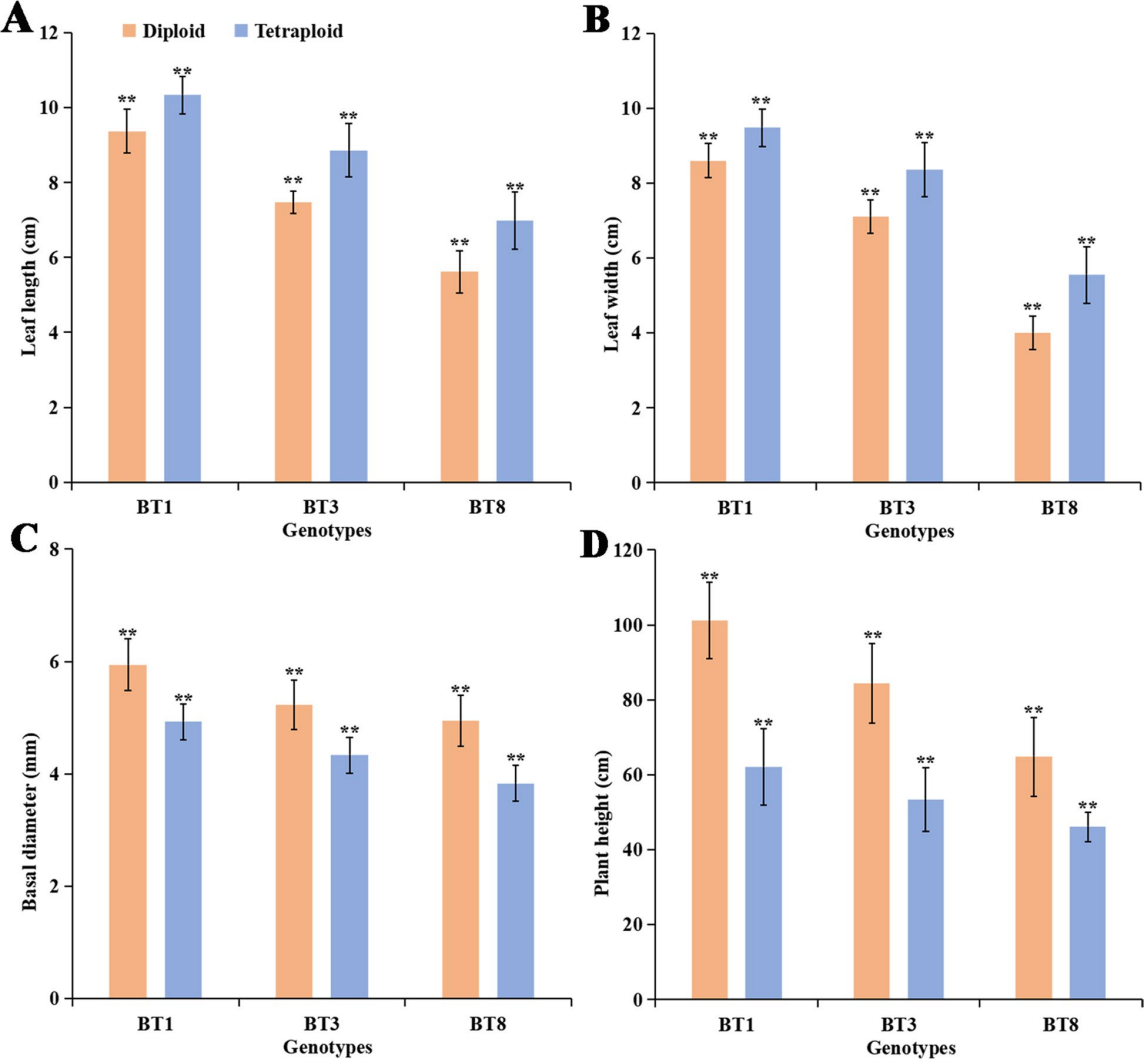
The leaf length **A**, leaf width **B**, basal diameter **C**, and plant height **D** in diploid and tetraploid plants of *P. hopeiensis*. The vertical bars show the standard error; the asterisk indicates significant differences between diploid and tetraploid plants (** *p* < 0.01)

The leaf length, leaf width, basal diameter, and plant height of diploid and tetraploid plants were further analyzed (Fig. 6). The mean leaf length and width in tetraploid plants of the three genotypes were significantly larger than that of diploids (Fig. 6A, B) (*t-*test, *P* < 0.01). However, the mean basal diameter and plant height in tetraploid plants of the three genotypes were significantly lower than that of diploids (Fig. 6C, D) (*t-*test, *P* < 0.01). The basal diameter of genotypes BT1, BT3, and BT8 decreased by 17.1, 17.2, and 22.5%, respectively. The plant height of genotypes BT1, BT3, and BT8 decreased by 38.6, 36.8, and 28.9%, respectively. These results indicate that the whole-genome duplication leads to tetraploids exhibiting a dwarf phenotype.

## Discussion

### Effects of genotype, 6-BA, TDZ, and IAA on shoot regeneration from leaf blades

Combining adventitious shoot regeneration from leaf blades with somatic chromosome doubling is a credible approach for creating new polyploids [24, 28, 55]. The shoot regeneration rate and the number of newly-regenerated shoots play a key role in affecting the polyploidization efficiency [24, 26]. Several studies have shown that adventitious shoot regeneration from leaf blades is affected by both the explant genotypes and the culture conditions, including the medium composition and addition of plant growth regulators [25, 56–58]. Li et al. [59] observed a significant correlation between genotype and leaf differentiation rate. Xu et al. [60] reported significant differences in the patterns of adventitious shoot initiation and callus development among different genotypes. In this study, we also found that the genotype had highly significant effects on the shoot regeneration rates and the number of shoots per explant, suggesting that the callus development of leaf blades among genotypes was inconsistent. These observations corroborate the findings of previous studies [60, 61].

In *Populus*, 6-BA, TDZ and IAA are widely used for adventitious shoot regeneration from leaf blades [56–58]. 6-BA and TDZ are the primary cytokinins that efficiently induce shoot regeneration and multiplication from leaf explants [24, 56]. However, high concentrations of 6-BA and TDZ may lead to the vitrification of the regenerated shoots. Previous studies documented that TDZ combined with 6-BA had a greater effect on the induction of adventitious shoots from leaf blades than 6-BA or TDZ used alone [26, 33, 56]. In the present study, 6-BA and TDZ had highly significant effects on shoot regeneration rates and the number of shoots produced per explant. The highest shoot regeneration rate and the number of shoots per explant were observed when MS medium contained 0.4 mg/L 6-BA and 0.015 mg/L TDZ, and no vitrification of regenerated shoots was observed under these conditions.

IAA is considered as a primary endogenous auxin. It plays an important role in regulating adventitious shoot regeneration from leaf blades [57]. Several previous studies have reported that the concentration and ratio of cytokinin and auxin are the main factors inducing shoot regeneration from leaf explants [56–58]. In general, low IAA concentrations can promote adventitious shoot elongation, thereby increasing the number of shoots per explant. However, high IAA concentrations may inhibit the induction and multiplication of adventitious shoots, leading to a reduced shoot regeneration rate [56–58]. Our results indicated that the shoot regeneration rates and the number of shoots per explant from *P. hopeiensis* leaf blades were obviously higher than those reported for *Populus deltoides* [57, 58], *Populus tremula* [62], and *Populus pseudo-simonii* [24].

### Effects of genotype, preculture duration, and colchicine treatment on tetraploid induction

Most research on tetraploid induction technology aims to establish an efficient procedure for inducing tetraploids. However, the most suitable treatment conditions may change with the nuances of the explant genotype, which may result in a lower rate of tetraploid production [63, 64]. Zhang et al. [33] and Xu et al. [25] found that leaf blades of different genotypes responded differently to colchicine treatment during tetraploid production. In our study, highly significant effects of genotype on the tetraploid induction rate were also observed. This might be due to variations in sensitivity to colchicine among the genotypes. Similar results have been reported in previous studies [33, 60, 63].

Colchicine is an antimitotic chemical mutagen that has been commonly used to generate new germplasm with higher ploidy levels in vascular plants [26, 28]. It can inhibit the polymerization of microtubules by binding to tubulin. Microtubules are a fundamental component of the cytoskeleton and involved in the formation of the mitotic spindle. Therefore, inhibiting polymerization inhibits chromosome separation during cell division and leads to the formation of a polyploid cell [63, 64]. Colchicine has been widely used to generate new tetraploids by somatic chromosome doubling in some tree species, such as *Populus* [25, 26, 65], *Platanus acerifolia* [31], *Pogostemon cablin* [32], *Robinia pseudoacacia* [29, 30], *Ziziphus jujuba* [27, 28], *Liquidambar styraciflua* [33], and *Lagerstroemia indica* [34]. In the present study, colchicine was applied to induce chromosome doubling by treating leaf blades, and 136 tetraploids of *P. hopeiensis* were successfully obtained. Hence, colchicine treatment of leaf blades is a promising way for creating new polyploids.

The preculture duration of leaf blades before colchicine treatment is an important factor affecting the formation of polyploids [32, 66, 67]. Cai and Kang [24] reported that no tetraploid plants were produced from leaf explants of *Populus pseudo-simonii* Kitag when there was no preculture before colchicine treatment. Cui et al. [28]. showed that the most effective preculture duration for inducing the tetraploids of leaf blades in diploid *Ziziphus jujuba* by colchicine treatment was 10 days. Zhang et al. [33] documented that the most effective preculture duration for inducing tetraploids with leaf blades via colchicine was 8 days for diploid *Liquidambar styraciflua*. However, in our study, the most suitable preculture duration for colchicine-induced tetraploidy of leaf blades in *P. hopeiensis* was 7 days. Our finding was inconsistent with the reported results in previous studies [24, 28, 33]. It is likely that the most suitable preculture duration depends on the species. Variations may also be due to the fact cell responses vary with cell cycle stages during treatment [68].

Polyploid formation was also highly correlated with the colchicine concentration and exposure time. In general, low colchicine concentrations or short exposure times are not effective for inducing polyploidy [27, 32]; high colchicine concentrations or long exposure time can result in leaf blade mortality due to the toxicity of colchicine [24, 55]. In our study, the highest tetraploid induction rate (21.2%) was observed when the leaf blades of *P. hopeiensis* were treated with 40 mg/L colchicine for 4 days, which is slightly higher than what has been reported in many other studies [24, 28]. This suggests that using a suitable colchicine concentration and exposure time will help improve the polyploid induction rate.

### Changes in ploidy-related morphological characteristics

Previous studies documented that the stomatal characteristics, such as stomatal length, stomatal width, and stomatal density, may be used as simple and efficient indicators to distinguish plant ploidy levels [29, 32, 54]. Furthermore, the stomata-based method is simple, virtually nondestructive, and does not require expensive instruments [28]. Here, we found that the stomatal length, width, and density of leaf explants in the induced tetraploids significantly differed from those observed in diploids. The stomatal length and width in tetraploids of the three genotypes were approximately twice that of diploid plants. However, the stomatal density in the tetraploids of the three genotypes was significantly lower than that of the diploids. Our results were consistent with the findings of previous studies [19, 55, 65, 69].

Polyploidy, or whole-genome duplication, provides genomic plasticity for functional divergence of duplicated genes, genomic and/or chromosomal recombination, transcriptome changes, and gene dosage effects, hence contributing to evolution [70–72]. An increase in DNA content and dosage effect of each gene, as is the case in polyploids, usually leads to an increase in organ size. Tsukaya [68] showed that the organs/bodies of the tetraploids in *Arabidopsis* were larger than the organs/bodies of diploids. Li et al. [29] documented that the leaf length and width were significantly larger in the tetraploids than in the diploids of *Robinia pseudoacacia*. In our study, tetraploids of *P. hopeiensis* produced larger leaf blades and had modified leaf blade morphology compared with diploids. However, the growth rate of the induced tetraploids in *P. hopeiensis* was also slower than that of the diploids. This suggests that gene dosage effects might increase the size of cells and organelles by altering gene expression, and this is generally accompanied by significant growth inhibition. Besides, the trend of trait variation after whole-genome duplication was consistent for all three genotypes of diploid *P. hopeiensis*. Hence, significant differences in the morphological characteristics between the diploids and the tetraploids may be a promising and stable tool for plant improvement. Additionally, tetraploid plants can be used as bridge parents to provide variation in chromosome number for the polyploid breeding program of *Populus* [11–13]. In future studies, we will focus on evaluating the growth and fertility of tetraploids and conduct interploid hybridation between the diploid and tetraploid *P. hopeiensis* individuals.

## Conclusions

Genotype, 6-BA, TDZ, and IAA had highly significant effects on the shoot regeneration rates and the number of shoots per explant. The suitable shoot regeneration medium from leaf blades of *P. hopeiensis* was MS medium supplemented with 0.4 mg/L 6-BA, 0.015 mg/L TDZ, and 0.1 mg/L IAA. The highest shoot regeneration rate and the number of shoots per explant were 96.7% and 11.7, respectively. The tetraploid induction rate varied highly significantly among different genotype, preculture duration, colchicine concentration, and colchicine exposure time. The optimal protocol for inducing tetraploids of different genotypes *P. hopeiensis* was leaf blades precultured for 7 days and then treated for 4 days by 40 mg/L colchicine, which can be applied to different genotypes of *P. hopeiensis*. The tetraploid induction rates of genotypes BT1, BT3, and BT8 were 21.2, 11.4, and 16.7%, respectively. A total of 136 tetraploids were determined by flow cytometry analysis and somatic chromosome counting. The stomatal length, width, and density of leaf blades significantly differed between diploid and tetraploid plants of the three genotypes. After six months of culture, the length and width of tetraploid leaf blades of the three genotypes were significantly larger than those of the diploids. However, the basal diameter and plant height were lower in tetraploids of the three genotypes than that of the diploids. In summary, the morphological features of tetraploid plants differed obviously and significantly from those of diploid plants.

## Methods

### Plant materials

The State Key Laboratory of Efficient Production of Forest Tree Resource, Beijing Forestry University, China, provided tissue culture sterile-rooted plantlets of the diploid *P. hopeiensis* genotypes (BT1, BT3, and BT8). These materials were transferred into rooting medium containing 0.2 mg/L IBA, 0.65% (w/v) agar, 3% (w/v) sucrose, and half-strength Murashige and Skoog (MS) medium [73] for rooting. All media were adjusted to pH 5.8–6.2, and subsequently autoclaved at 121℃ for 15 min. All cultures were illuminated by fluorescent tubes of 2,000 lx with a 14 h photoperiod in a growth chamber at 25 °C.

### Shoot regeneration of leaf blades

Fully expanded leaf blades from 30-day-old sterile-rooted plantlets were partially transected in two locations crossing the main vein so the leaf remained intact; they were then transferred to MS basal medium containing different concentrations of 6-BA (0.2, 0.3, and 0.4 mg/L), TDZ (0.005, 0.01, and 0.015 mg/L) and IAA (0.05, 0.1, and 0.2 mg/L). The effects of 6-BA, TDZ, and IAA on shoot regeneration were studied utilizing an orthogonal experimental design (Table 1). After 45 days in culture, the number of regenerated leaf blades and regenerated shoots per treatment was recorded to determine the shoot regeneration rates and the number of shoots per explant. The experiments were replicated three times with 10 explants per treatment for each genotype.

### Tetraploid induction by colchicine treatment

The leaf blades were partially transected in two locations crossing the main vein, so the leaf remained intact. The leaf blades were then transferred to MS shoot regeneration medium containing 0.4 mg/L 6-BA, 0.015 mg/L TDZ, and 0.01 mg/L IAA, 0.65% (w/v) agar, and 3% (w/v) sucrose for 5, 6, and 7 days. Explants were then immersed in the same liquid shoot regeneration medium with filter-sterilized colchicine at concentrations of 20, 30, and 40 mg/L and treated in the dark for 2, 3, and 4 days, respectively. The 27 treatments are listed in Table 3. Following colchicine treatment, the explants were transferred to fresh solid shoot regeneration medium without colchicine after being rinsed three times with sterile distilled water. The experiments were replicated three times with 10 explants per treatment for each genotype.

After culture for 45 days, single shoots regenerated from the injuries of leaf blades were excised and transferred to the rooting medium. The number of regenerated shoots and tetraploids per explant were recorded to determine tetraploid induction rates. Subsequently, every 30 days, apical buds excised from the sterile-rooted plantlets were transferred to fresh solid rooting medium for culture.

### Ploidy level determination

The ploidy level of all regenerated plantlets arising from leaf blades by colchicine treatment was preliminarily determined by flow cytometry analysis using the same procedures, reagents, and cytometers as those used by Wu et al. [26]. The standard peak of leaf blades from untreated diploid plants was set to appear at channel 50 relative fluorescent intensity. Therefore, the regenerated plantlets were considered as tetraploids when their peaks appeared at channel 100 relative fluorescent intensity.

The putative tetraploid plants were further confirmed by chromosome counting. Stem tips were harvested from 25-day-old sterile-rooted plantlets and pretreated in a saturated solution of paradichlorobenzene for 2–4 h. Then, they were washed three times with distilled water and fixed in fresh Carnoy’s solution (acetic acid: ethanol, 1:3) for 24 h at 4℃. The fixed samples were dissociated in 38% HCl for 25 min and washed with distilled water three times for 15 min. Subsequently, the dissociated samples were chopped on a glass slide in a drop of carbol-fuchsin solution and covered with a coverslip. The samples were observed and photographed under a 100 × oil lens using an Olympus BX 51 microscope. The ploidy number was verified from 10 randomly sampled cells of each sample.

### Analysis of stomatal and morphological characteristics

Mature leaf blades from 30-day-old sterile-rooted plantlets were used for the analysis of stomatal characteristics. Six tetraploid and diploid plantlets (three each) were sampled for observation of stomata. Stomatal length, width, and density from tetraploid and diploid leaf blades were measured following previously described methods [55].

After 30 days of rooting culture, 45 diploid and 45 tetraploid rooted plantlets with prolific root systems from each genotype were randomly sampled, subsequently transplanted into plastic pots that were filled with potting media (turf soil: vermiculite: perlite, 2:1:1), and placed in a greenhouse. All the plants for the growth experiment were randomly planted and placed to reduce environmental influence. After six months of culture, the leaf length, leaf width, basal diameter, and plant height of diploid and tetraploid plants of the three genotypes were measured. Plants tested in the greenhouse were 15 replicates per genotype and ploidy level, and these plants were randomly selected.

### Statistical analysis

Statistical analyses were implemented using IBM SPSS Statistics 20.0 software (IBM Inc., New York, USA). Before analysis of variance, percentage data were converted to proportions (p/100), and then arcsine square root-transformed to satisfy the heteroscedasticity assumptions. The univariate GLM analyses were used to analyze variation in the shoot regeneration rates, the number of shoots per explant, and tetraploid induction rates. The LSD multiple comparison tests were performed to assess the significance of differences between treatments; the threshold for statistical significance in these tests was *P* < 0.05. Two-sample *t*-tests were conducted to evaluate whether diploid and tetraploid plants significantly differed in stomatal length, stomatal width, stomatal density, leaf length, leaf width, basal diameter, and plant height.

## Data Availability

All data generated or analysed during this study are included in this published article.

## Abbreviations

MS: Murashige and Skoog basal medium
6-BA: 6-Benzylaminopurine
TDZ: Thidiazuron
IAA: Indole-3-acetic acid
IBA: Indole-3-butyric acid
GLM: General linear model
LSD: Least significant difference
